# Targeting GD2-positive glioblastoma by chimeric antigen receptor empowered mesenchymal progenitors

**DOI:** 10.1038/s41417-018-0062-x

**Published:** 2018-11-22

**Authors:** Giulia Golinelli, Giulia Grisendi, Malvina Prapa, Marco Bestagno, Carlotta Spano, Filippo Rossignoli, Franco Bambi, Iacopo Sardi, Monica Cellini, Edwin M. Horwitz, Alberto Feletti, Giacomo Pavesi, Massimo Dominici

**Affiliations:** 1 Department of Medical and Surgical Sciences for Children and Adults, Division of Oncology, University-Hospital of Modena and Reggio Emilia, Modena, Italy; 2 Rigenerand Srl, Medolla, Modena, Italy; 3 International Centre for Genetic Engineering and Biotechnology, Trieste, Italy; 4 Cell Factory Meyer, “A. Meyer” University Children’s Hospital, Florence, Italy; 5 Neuro-Oncology Unit, Department of Pediatric Oncology, Meyer Children’s University Hospital, Florence, Italy; 6 Onco-Hematology Pediatric Unit, Department of Medical and Surgical Sciences for Mothers, Children and Adults, University-Hospital of Modena and Reggio Emilia, Modena, Italy; 7 Aflac Cancer and Blood Disorders Center, Children’s Healthcare of Atlanta and Emory University Department of Pediatrics, Atlanta, GA USA; 8 Department of Neurosurgery, NOCSAE Hospital of Modena, Modena, Italy

**Keywords:** Targeted therapies, CNS cancer, Drug development

## Abstract

Tumor targeting by genetically modified mesenchymal stromal/stem cells (MSCs) carrying anti-cancer molecules represents a promising cell-based strategy. We previously showed that the pro-apoptotic agent tumor necrosis factor-related apoptosis-inducing ligand (TRAIL) can be successfully delivered by MSCs to cancer sites. While the interaction between TRAIL and its receptors is clear, more obscure is the way in which MSCs can selectively target tumors and their antigens. Several neuroectoderm-derived neoplasms, including glioblastoma (GBM), sarcomas, and neuroblastoma, express high levels of the tumor-associated antigen GD2. We have already challenged this cell surface disialoganglioside by a chimeric antigen receptor (CAR)-T cell approach against neuroblastoma. With the intent to maximize the therapeutic profile of MSCs delivering TRAIL, we here originally developed a bi-functional strategy where TRAIL is delivered by MSCs that are also gene modified with the truncated form of the anti-GD2 CAR (GD2 tCAR) to mediate an immunoselective recognition of GD2-positive tumors. These bi-functional MSCs expressed high levels of TRAIL and GD2 tCAR associated with a robust anti-tumor activity against GD2-positive GBM cells. Most importantly, the anti-cancer action was reinforced by the enhanced targeting potential of such bi-functional cells. Collectively, our results suggest that a truncated anti-GD2 CAR might be a powerful new tool to redirect MSCs carrying TRAIL against GD2-expressing tumors. This affinity-based dual targeting holds the promise to combine site-specific and prolonged retention of MSCs in GD2-expressing tumors, thereby providing a more effective delivery of TRAIL for still incurable cancers.

## INTRODUCTION

Human mesenchymal stromal/stem cells (MSCs) are considered pivotal players in cellular therapy. Their isolation easiness from different sources and their capacity to be engineered by viral vectors make them cellular vehicles to deliver anti-tumor agents [1]. As putative tumor stroma cell precursors, MSCs can localize within tumors by promising but still unclear mechanisms of action [2]. A homing ability of MSCs has been reported towards several cancers including glioma, breast, colon, ovarian, pancreatic, and lung carcinomas, among many other tumors. Once to the tumor site, they can offer a prolonged and concentrated local delivery of therapeutic molecules, reducing a non-selective targeting to possibly improve efficacy of standard treatments [3].

Glioblastoma (GBM) is the most common primary malignant brain tumor in human. Despite considerable advances in therapies, GBM remains one of the most challenging diseases [4]. The invasive nature of GBM is the major reason underlying failure of surgical resection alone or in association with standard radiation and temozolomide-based chemotherapy [5]. Novel strategies based on oncolytic viral vectors have difficulties in reaching metastatic niches from the main tumor burden [6, 7].

New effective tools are needed to specifically target cancer cells within a context characterized by a high local metastatic potential [8]. It has been demonstrated that MSCs can be primarily attracted by GBM while sparing healthy brain in such a way to become a tumor-specific drug delivery system [9]. Recently, few strategies have been envisioned to attain an effective and localized MSCs delivery to GBM extending the therapeutic effect and limiting off-target toxicities [10–12]. Despite MSCs tropism for tumor microenvironment, the engraftment and retention of MSCs in tumor still remains an issue to be addressed [13].

Therefore, parallel to homing, particular attention is needed on how to improve MSC affinity for the tumor site in order to achieve tumor-specific targeting and retention [14]. Much of the groundwork for affinity-based targeting approaches has been established in the field of viral gene delivery and in adoptive immunotherapy. The highest binding abilities have been obtained using antibodies, TCR, and chimeric antigen receptor (CAR) [15].

This targeting takes advantage of surface molecules uniquely or highly expressed by tumor cells. Regarding GBM, it has been demonstrated that a variety of GBM lines and primary biopsies commonly express high levels of disialoganglioside GD2 antigen, similarly to several pediatric and adult cancers [16, 17]. GD2 levels increase in GBM compared to the surrounding normal brain, being a relatively minor component of the normal central nervous system (<4% of total gangliosides). For this reason, GD2 becomes an attractive clinical target for GBM and other brain tumors [16].

In recent years, our group developed T cells expressing anti-GD2 CAR [18]. CAR affinity domain, represented by the single-chain variable fragment (scFv) of an immunoglobulin against a specific tumor marker, redirects the killing functions of immune cells to the defined target upon recognition, thereby enhancing the retention to the tumor site [19]. In a parallel project, adipose (AD)-MSCs were modified by viral vectors in order to constantly express a variant of the potent anti-tumor ligand tumor necrosis factor-related apoptosis inducing ligand (TRAIL) allowing a local effective killing in a variety of cancers [20–23].

Taking inspiration from CAR T cells as successful examples for the specific recognition and binding to target cells, we originally further optimized tumor-localizing potential of TRAIL-expressing MSCs. Thus, we combined anti-GD2-targeted strategy where TRAIL is expressed on AD-MSCs together with a truncated form of a previously developed anti-GD2 CAR (GD2 tCAR) [18]. This GD2 tCAR lacks the intracellular signaling domain while preserving the transmembrane region and the extracellular anti-GD2 binding moiety. Owing to this immunoselective GD2-based targeting, MSCs carrying TRAIL have been specifically redirected towards GD2-expressing tumors while improving cell-to-cell interaction. To the best of our knowledge, this study is the first report on the genetic modification of therapeutic TRAIL MSCs to express an artificial receptor (AR) against GD2. TRAIL MSCs killing function coupled with GD2 tCAR affinity-based tumor targeting could be advantageous to improve therapeutic delivery of TRAIL molecule for the treatment of still deadly GD2-positive malignancies.

## Materials and methods

### Cell culture and maintenance

Human GBM cell lines T98G, U87MG, and A172 are a kind gift from Dr. Franco Bambi (Meyer Children’s Hospital, Florence, Italy). The primary C3c GBM cell line was obtained from a patient affected by GBM (female, IV grade) and collected at the Department of Neurosurgery, Nuovo Ospedale Civile S. Agostino Estense, Modena, Italy. Tissue procurement was approved by the local Ethical Committee (#3600/27 September 2017). T98G and U87MG cell lines were cultivated in minimum essential medium (MEM) Eagle's with Earle's salts (Euroclone, Milan, Italy). A172 were maintained in Dulbecco’s modified Eagle's medium (DMEM; Gibco, Thermo Fisher Scientific, Waltham, MA, USA). The primary C3c GBM cell line was kept in DMEM/F-12 (Gibco). Media were supplemented with 10% fetal bovine serum (Carlo Erba Reagents Srl, Cornaredo, Italy), 1% l-glutamine (200 mM) (BioWhittaker, Lonza, Verviers, Belgium), and 1% penicillin–streptomycin (pen/strep; 10^4^ UI/ml and 10 mg/ml; Carlo Erba Reagents Srl). Human AD-MSCs were obtained as previously described [20]. After isolation, cells were grown in α-MEM (Gibco) containing 2.5% platelet lysate (Modena Policlinic Blood Bank, Modena, Italy), 1% l-glutamine, 0.5% ciprofloxacin (Fresenius Kabi Italia srl, Verona, Italy), and 0.2% heparin (Sigma-Aldrich, Saint Louis, MO, USA). Cells were incubated and maintained within a controlled atmosphere, 5% CO_2_, and temperature of 37 °C. Authentication of T98G, U87MG, and A172 cell lines has been recently performed by the Leibniz Institute DSMZ - German Collection of Microorganisms and Cell Cultures GmbH, Braunschweig, Germany.

### Viral vectors and AD-MSC transduction

**Fig. 1:**
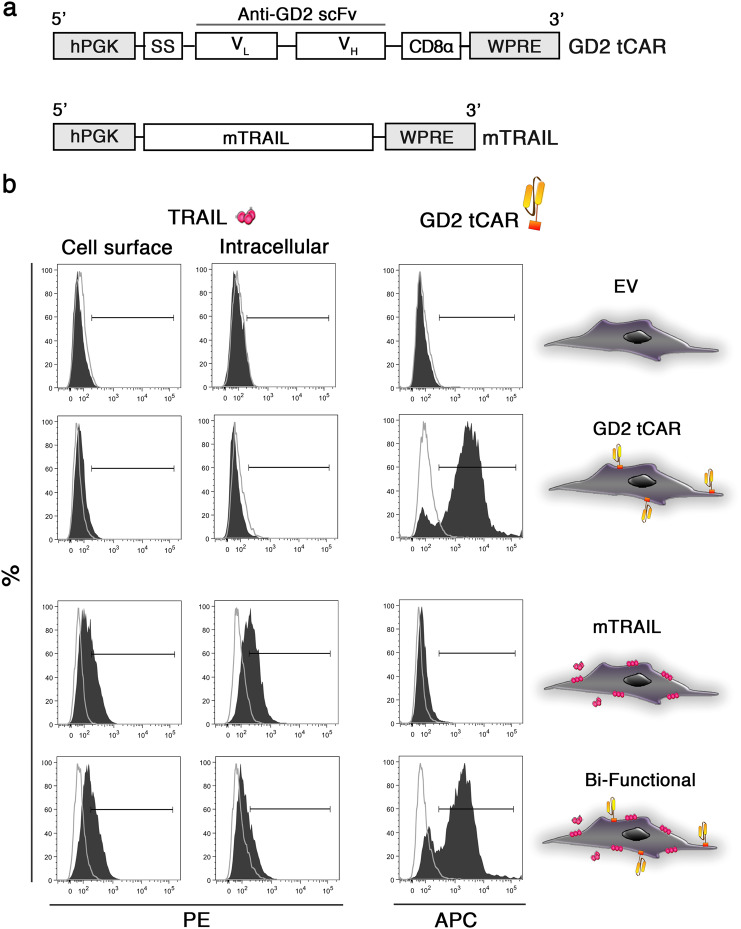
MSCs are effectively transduced with GD2 tCAR and/or TRAIL encoding vectors. **a** Schematic presentation of the GD2 tCAR expression construct (upper) in which the IgM-derived anti-GD2 scFv was fused with the human CD8α-derived hinge-transmembrane domain and cloned between PGK and WPRE lentiviral regulatory elements, the mTRAIL expression construct (lower) carrying the full-length human TRAIL gene between PGK and WPRE. **b** FACS analysis of TRAIL and GD2 tCAR expression on empty vector-transduced MSCs, EV MSCs (row 1), GD2 tCAR MSCs transduced with GD2 tCAR vector (row 2), mTRAIL MSCs transduced with vector coding for mTRAIL (row 3), and bi-functional MSCs coding for both GD2 tCAR and mTRAIL (row 4)

We recently generated a CAR able to bind the disialoganglioside GD2 for an immunotherapy approach against GD2-expressing cancers [18]. A truncated form of that CAR lacking the signaling domains 4-1BB (CD137) and CD3-ζ has been introduced into AD-MSCs (Fig. 1a, row 1). Truncated anti-GD2 CAR (GD2 tCAR) gene is composed by an anti-GD2 scFv from a murine antibody of immunoglobulin M (IgM) class linked to a portion of CD8, the CD8α hinge-transmembrane domain. The GD2 tCAR expression cassette was cloned into the multiple cloning site of the lentiviral vector pCCL PGK WPRE, using *Bam*HI and S*al*I restriction enzymes. Properly sequenced full-length human TRAIL gene has been cloned (*Xma*I/*Sal*I) into the lentiviral vector pCCL PGK WPRE to gene modify AD-MSCs for the surface expression of membrane-bound TRAIL (mTRAIL) molecule (Fig. 1a, row 2). AD‐MSC transductions were performed as previously reported [23]. The transduced AD-MSC lines were defined as empty vector (EV MSCs), GD2 tCAR vector (GD2 tCAR MSCs), vector carrying mTRAIL alone (mTRAIL MSCs) and bi-functional MSCs coding for both GD2 tCAR and mTRAIL.

### Fluorescence-activated cell sorting

TRAIL presence on AD-MSCs was assayed by a phycoerythrin (PE)-conjugated anti-TRAIL antibody (BioLegend, San Diego, CA, USA). Intracellular staining on transduced MSCs was performed with Becton Dickinson Cytofix/Cytoperm Kit (BD, Franklin Lakes, NJ, USA). To detect GD2 tCAR expression on AD-MSCs, anti-idiotypic antibodies were raised as previously described [18, 24]. AD-MSCs were incubated with anti-idiotype mice sera followed by allophycocyanin (APC)-conjugated goat anti-mouse secondary antibody (BD). TRAIL receptors expression on GBM cell lines was tested by fluorescence-activated cell sorter (FACS), as well. Tumor cell lines were stained by PE-conjugated anti-TRAIL-R1/DR4 and anti-TRAIL-R3/DcR1, and APC-conjugated anti-TRAIL-R2/DR5 and anti-TRAIL-R4/DcR2 (BioLegend). To evaluate GD2 antigen expression, tumor cell lines were stained with primary unconjugated mouse anti-human GD2 (BD) and then with APC goat anti-mouse Ig (APC Goat Anti-Mouse Ig polyclonal multiple adsorption; BD). In all the experiments, corresponding isotype-matched antibodies were used as negative controls (provided by BD and BioLegend). Data were collected using FACS Aria III flow cytometer (BD) and analyzed using the FACS Diva software (BD).

### Dose–response rhTRAIL

Tumor cell lines were seeded in 96-well cell culture plate at 6000 cells/cm^2^. After 12 h, different concentrations (10 ng, 50 ng, 100 ng, 500 ng, and 1000 ng/ml) of recombinant human TRAIL (rhTRAIL) (Peprotech, London, UK) were added in the culture media. Proliferation rate was assessed after 24 h by the colorimetric assay CellTiter 96 AQueous One Solution Cell Proliferation Assay (Promega, Madison, WI, USA). Alternatively, tumoricidal activity of rhTRAIL was evaluated by supravital propidium iodide (PI; Sigma-Aldrich) staining after 24 h by FACS.

### Cytotoxicity assays

Tumor cell lines were seeded in 12-well cell culture plate at 6000 cells/cm^2^. After 12 h, all gene-modified MSCs were labeled with carboxyfluorescein succinimidyl ester (CFSE) cell tracker (Molecular Probes, Eugene, OR, USA) and added at different target-to-effector (T:E) ratios (1:1, 1:2, and 1:5). The tumoricidal activity of MSC-expressing TRAIL was evaluated by PI or Annexin V (BD)/PI staining after 12, 24, and 48 h by FACS gating on CFSE-negative cells and PI-positive or Annexin V/PI-double-positive events. Soluble rhTRAIL at 1 μg/ml was introduced as a positive control.

### Cell-to-cell interaction assays

The cell-to-cell interaction assay was established by the selective target cell recognition mediated by GD2 tCAR-functionalized MSCs against GBM cell lines that markedly differ for GD2 expression levels. A monocistronic murine stem cell virus-derived retroviral vector encoding for the red fluorescent protein DsRed was used to stably transduce GBM cell lines (T98G, U87MG, and A172) and to distinguish them from MSCs into the cell-to-cell interaction assay. Retrovirus production was performed by FLYRD18 packaging cell line, as published [20]. The primary C3c GBM line was labeled by CellTracker Deep Red Dye (Thermo Fisher Scientific).

Similarly, MSCs were labeled with the CFSE fluorescent dye according to the reagent’s manual. A cell-to-cell interaction assay was performed as described. CFSE-labeled MSCs were seeded into a 12-well cell culture plate at a high density (100,000 cells/well) to create a confluent monolayer in 12 h. To test cell-to-cell interactions, a suspension of DsRed (or Deep Red for primary C3c GBM) tumor cells (70,000 cells per well) were added to the MSC monolayer. After 1 h and half incubation time, media were removed and the coculture were washed twice in phosphate-buffered saine (PBS) and shaken for 3 min in order to lose weakly adherent tumor cells. An incubation time of 20 min has been identified as optimal for the primary C3c GBM line, as this line has a greater basal adherence to MSCs in comparison to that of GBM lines. The cells were then collected by trypsinization and suspended in a constant volume of PBS. The absolute number of MSC–tumor cell aggregates was quantified by FACS considering the CFSE/DsRed (or CFSE/Deep Red)-double-positive population in a constant time frame of 60 s. Data were expressed comparing the number of MSC–tumor cell aggregates acquired for all the conditions versus EV MSCs.

A cell-to-cell interaction cytotoxicity assay was further challenged to detect the bi-functional MSC-specific cytotoxic effect against GD2-positive GBM cells at an early time point of 7 h incubation. The early cell death in MSC-GBM cell aggregates was evaluated by Annexin V staining and FACS analysis gating on CFSE/DsRed-double-positive conglomerates recording a fixed number of events, for all the conditions. Data were expressed as the percentage of Annexin V-positive MSC-GBM cell aggregates. Anti-idiotype mice sera against GD2 tCAR were further used to block the GD2 tCAR-mediated interaction between bi-functional MSCs and GD2-expressing GBM cells and the effect on apoptosis was investigated. Briefly, MSCs were incubated with a 1:20 dilution of mice sera for 40 min at 37 °C, 5% CO_2_, and then used to set up a cell-to-cell interaction assay as described above. The percentage of Annexin V-positive MSC-GBM cell aggregates at a 7 h incubation was analyzed by FACS.

### Statistics

Analyses were performed using Excel 2016 program (Microsoft Inc., Redmond, WA, USA). Data are expressed as mean ± SD. A *p* value of ≤ .05 from two-tailed Student’s *t* test was considered statistically significant. Normal distribution of data has been tested using Shapiro–Wilk normality test. For the assays, each experimental group was assayed at least twice in triplicate.

## Results

### Engineered bi-functional MSCs express GD2 tCAR and deliver TRAIL

Co-expression of GD2 tCAR together with mTRAIL in MSCs was obtained by lentiviral vector transduction. The presence of mTRAIL and GD2 tCAR molecules was verified by FACS on transduced MSCs (Fig. 1b). TRAIL and GD2 tCAR were undetectable on EV MSCs (Fig. 1b, row 1), while GD2 tCAR was exclusively revealed in 79 ± 7% of GD2 tCAR MSCs (Fig. 1b, row 2). As expected, TRAIL presence was confirmed on 95 ± 8% of mTRAIL MSCs (55 ± 7% on cell membrane and 40 ± 15% in the cytoplasm; Fig. 1b, row 3). Bi-functional MSCs expressed TRAIL together with the GD2 tCAR. In particular, TRAIL was detected in 71 ± 4% of bi-functional MSCs (49 ± 7% on cell membrane and 22 ± 3% in cytoplasm), and 65 ± 17% of bi-functional MSCs were also positive for GD2 tCAR (Fig. 1b, row 4). These findings demonstrate that high levels of GD2 tCAR on bi-functional MSCs do not affect TRAIL production, underlining the feasibility of our dual-targeting approach.

### GBM cells differentially express GD2, have high expression of TRAIL receptor DR5, and are sensitive to rhTRAIL

**Fig. 2:**
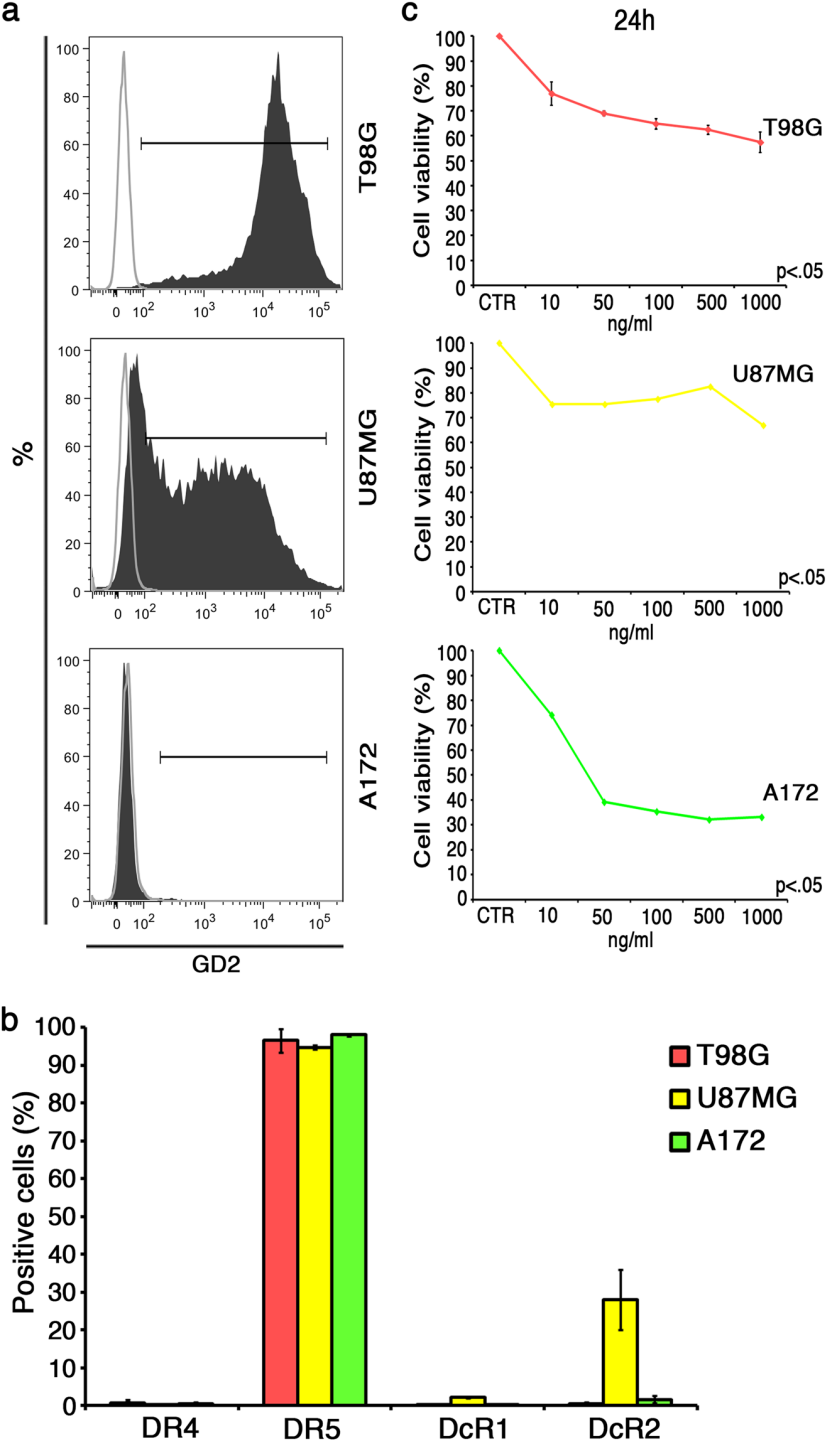
GBM target cells characterization. **a** Representative histograms showing GD2 expression, dark gray curve, on human T98G (97 ± 1%), U87MG (57 ± 13%), and A172 (2 ± 1%) GBM cell lines by FACS. APC-conjugated secondary Ab was used as isotype and represented by light gray line. **b** Expression of both agonistic (DR4 and DR5) and decoy (DcR1, DcR2) TRAIL receptors on GBM cell lines by FACS. **c** Sensitivity of GBM tumor cells to apoptosis induced by recombinant human TRAIL (rhTRAIL). T98G cell viability by supravital propidium iodide (PI) staining, U87MG and A172 cell viability by MTS assay after 24 h of rhTRAIL treatment at different doses in comparison with untreated control (CTR). *p* < .05 by Student’s *t* test between the highest rhTRAIL dose (1000 ng/ml) and untreated CTR, for all GBM lines. Data are expressed as mean ± SD

Having generated the effectors, in order to challenge our cell therapy approach against three different GBM cell lines, we began testing both GD2 and TRAIL receptors, as predictive factor for affinity-based targeting and TRAIL sensitivity. FACS analyses revealed that the three selected GBM cell lines differ for GD2 expression (Fig. 2a). Specifically, we could distinguish GBM cell lines in the GD2 highly positive T98G (97 ± 1%), the GD2 middle-positive U87MG (57 ± 13%), and the GD2-negative A172 (2 ± 1%). Similarly to T98G, the primary C3c GBM line expressed high levels of GD2 (97%; not shown).

On TRAIL receptors expression (Fig. 2b), FACS analyses revealed high levels of DR5 (≥95%) and negligible expression of DR4 for all lines. Decoy receptor DcR1 was undetectable in all cell lines (<2%), whereas DcR2 was positive for U87MG (28 ± 8%) with very low presence on A172 (2 ± 1%) and negative for T98G (0.5 ± 0.5%). In addition, the primary C3c GBM line highly expressed the DR5 (89%), while it was negative for DR4 (0.1%), DcR1 (0.1%), and DcR2 (1.9%) (not shown). Dose–response tests were introduced to verify rhTRAIL sensitivity on GBM cell lines (Fig. 2c). Despite similar levels of DR5, GBM lines demonstrated differences in rhTRAIL responsiveness with A172 being the most sensitive (33 ± 0.02% cell viability; *p* < .05) and U87MG the lowest one (67 ± 0.02% cell viability; *p* < .05), after 24 h at the highest rhTRAIL dose (1 μg/ml).

### Bi-functional MSCs induce in vitro apoptosis in GBM cells

**Fig. 3:**
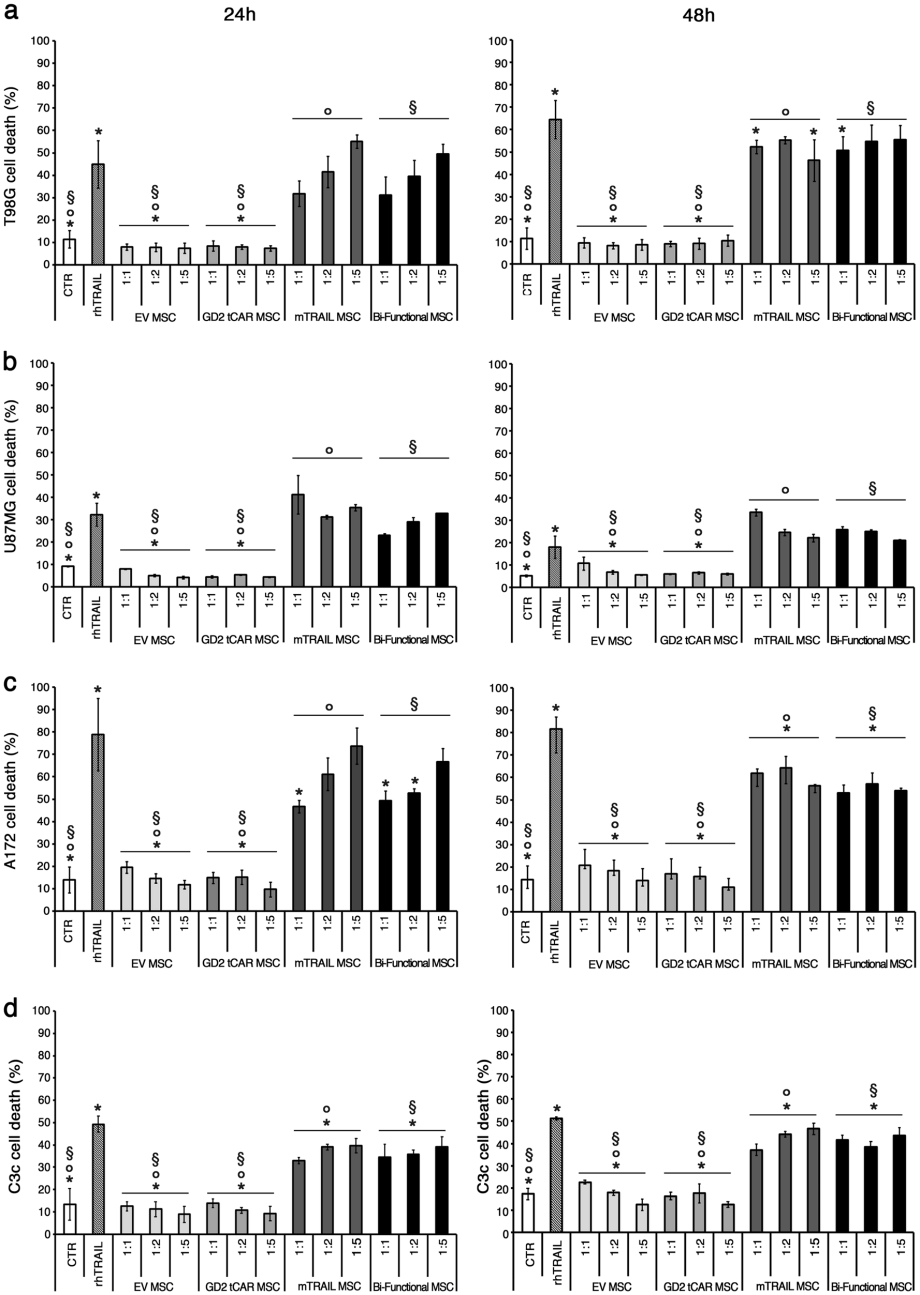
Bi-functional MSCs exert in vitro cytotoxicity on target GBM cell lines. **a** In vitro impact of bi-functional MSCs against **a** T98G, **b** U87MG, **c** A172 GBM lines, and **d** primary C3c GBM cells testing multiple target-to-effector ratios (1:1, 1:2, and 1:5). Tumor cell death by supravital propidium iodide (PI) for T98G, A172, and C3c and by Annexin V/PI staining for U87MG after 24 h (left column) and 48 h (right column). Recombinant human TRAIL (rhTRAIL, 1μg/ml) was used as a positive control of cell death, while tumor cell lines alone as a negative control (CTR). Reported *p* values regard multiple comparisons among mTRAIL MSCs and bi-functional MSC conditions versus control groups represented by EV MSCs, GD2 tCAR MSCs, rhTRAIL, or CTR. For T98G, *p < .05, °*p* < .01, ^§^*p* < .01; for U87MG, *p < .05, °*p* < .05, ^§^*p* < .05; for A172, *p < .05, °*p* < .01, ^§^*p* < .05; for C3c, *p < .05, °*p* < .001, ^§^*p* < .00. All *p* values have been calculated by Student’s *t* test. Data are expressed as mean ± SD

Having proved the sensitivity of GBM lines to TRAIL pro-apoptotic effect, we performed cocultures to verify the effectiveness of our cell therapy strategy based on genetically modified bi-functional MSC-expressing mTRAIL and GD2 tCAR. The killing potential mediated by bi-functional MSCs was assessed testing different time points (24 and 48 h) and multiple target to effector ratios (1:1, 1:2, and 1:5 T:E ratios; Fig. 3). The green fluorescence of CFSE-labeled MSCs allowed a clear distinction between target (CFSE-negative) and effector cells (CFSE-positive) by FACS. Gating on CFSE-negative and PI-positive tumor cells, we were able to specifically quantify the percentage of tumor cell death. The bi-functional MSCs cytotoxic effect was also challenged comparing the impact induced by rhTRAIL (1 μg/ml) or mTRAIL MSCs, whereas EV MSCs and GD2 tCAR MSCs were used as negative controls. Starting from 24 h, all tested GBM cell lines displayed a significant sensitivity to TRAIL delivered by MSCs (Fig. 3, left column). A172 appeared to be the most sensitive line (up to 73 ± 8% cell death, with the highest mTRAIL MSC dose; Fig. 3c, left column), whereas U87MG appeared to be less sensitive to both rhTRAIL and mTRAIL MSCs (32 ± 5% and 41 ± 8% cell death, respectively; Fig. 3b, left column). At 1:5 ratio, bi-functional MSCs were able to exert a robust cytotoxic effect in T98G and A172 lines (50 ± 4% and 67 ± 6% cell death respectively, *p* < .001), provoking a mortality rate comparable to mTRAIL MSCs or rhTRAIL (*p* > .05, Fig. 3a, c, respectively, left column). The cytotoxicity mediated by bi-functional MSCs increased according to the T:E ratios for T98G and A172 GBM lines at 24 h (*p* < .05). The GBM cell apoptosis by bi-functional MSCs was further confirmed at 48 h (Fig. 3, right column) with a similar trend of the 24 h. Once again, T98G and A172 lines appeared to be particularly responsive to both mTRAIL MSCs and bi-functional MSCs (*p* < .05; Fig. 3a, c, respectively, right column). In particular, at 48 h bi-functional MSCs were able to induce tumor cell death in T98G (55 ± 6%) and A172 lines (57 ± 5%) with levels that remained stable regardless of effectors number, unlike to what observed at 24 h (*p* > .05). For all tested culture conditions and time points, cocultures of GBM cell lines with EV MSCs or GD2 tCAR MSCs did not impact tumor cell survival.

The killing by bi-functional MSCs was further challenged against the primary C3c GBM line (Fig. 3d). Bi-functional MSCs were able to exert a cytotoxic effect, provoking the highest tumor mortality (44 ± 4%) at 1:5 T:E ratio at 48 h, comparable to mTRAIL MSCs (*p* > .05). Similarly to what observed for cell lines, EV MSC or GD2 tCAR MSC cocultures did not impact cell survival on primary C3c cells.

These in vitro data outline a significant, although variable, sensitivity of GBM towards MSCs delivering TRAIL. In particular, we demonstrated that the simultaneous expression of pro-apoptotic TRAIL ligand and GD2 tCAR does not affect the killing by engineered MSC, thus prompting our dual-targeting approach against GD2-postitive GBM.

### GD2 tCAR functionalization improves the interaction with GD2-expressing GBM cells

We hypothesized that MSCs surface functionalization with the GD2 tCAR is able to force interaction between MSCs and GBM cells expressing GD2. To investigate the new properties acquired by bi-functional MSC, we tested their ability to bind GBM in a cell-to-cell interaction assay. For this purpose, MSCs were labeled by CFSE, to acquire a green fluorescence; conversely, GBM cell lines were stably infected with viral vector coding for DsRed gene to exhibit a red fluorescence, while the primary C3c GBM line was labeled by the CellTracker Deep Red dye.

By these different labeling strategies, MSCs and GBM cells were clearly distinguishable by FACS and their aggregates appeared as double positive for both CFSE and DsRed (or Deep Red) fluorescence. In a cell-to-cell interaction assay GBM cells were added to CFSE-labeled MSC monolayers and left in interaction for 1.5 h (or 20 min for primary C3c cells).

**Fig. 4:**
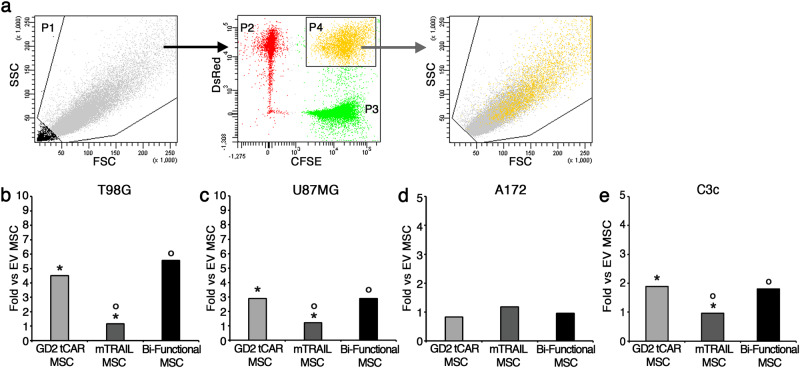
GD2 tCAR on MSCs surface increases binding to GD2-expressing GBM cells in vitro by a cell-to-cell interaction assay. **a** Gating strategy for the analysis of the absolute number of MSC-GBM cell aggregates after 1.5 h incubation time. Gate 1: forward scatter area (FSC) and side scatter area (SSC) were used to enrich for intact cells (P1). Gate 2: CFSE staining (P3) and DsRed (or Deep Red) fluorescence (P2) recorded in the logarithmic scale were used to identify MSC-GBM cell aggregates appeared to be CFSE/DsRed (or CFSE/Deep Red) double positive (P4). Backgating in P1 population to identify MSC-GBM cell aggregates by morphological parameters. To quantify the absolute number of aggregates, for all tested conditions, we considered the number of cellular events acquired into the CFSE/DsRed (or CFSE/Deep Red) double-positive gate (P4) over 60 s. **b**–**e** Number of MSC-GBM cell aggregates reported as fold of all conditions versus EV MSCs, for all GBM cell lines and primary C3c GBM cells. GD2 tCAR MSCs and bi-functional MSC bound GD2-positive cells, T98G (**b**), U87MG (**c**), and C3c (**e**) greater than EV MSCs and mTRAIL MSCs (*^,^°*p* < .05). For A172 negative line (**d**), no differences in aggregates formation were found between the MSC types. *^,^°*p* < .05 by Student’s *t* test. Data are expressed as mean ± SD

After sequential washing to lose weakly interactors, cells were collected by trypsinization and analyzed by FACS to determine the absolute number of MSC-GBM cell aggregates, visualized as CFSE/DsRed (or CFSE/Deep Red) double-positive events. The related gating strategy is described in Fig. 4a. Cells were first gated on forward scatter area (FSC) and side scatter area (SSC) to exclude debris (P1), then DsRed (or Deep Red)-GBM cells (in red; P2) and CFSE-MSCs (in green; P3) were visualized in a CFSE/DsRed (or CFSE/Deep Red) dot plot to finally gate the double-positive events, corresponding to the MSC-GBM cellular aggregates and represented as yellow dots in the upper right quadrant (P4). A backgate of CFSE/DsRed (or CFSE/Deep Red) double-positive population in P1 confirmed the greater size and complexity of MSC-GBM cells conglomerates. The number of aggregates generated by cell-to-cell interaction of GBM cells with either GD2 tCAR MSCs, or bi-functional MSCs or mTRAIL MSCs were normalized to EV MSC-GBM cell aggregate numbers and data displayed as fold, for all GBM lines tested (Fig. 4b). Very interestingly, the fold strictly correlated with both GD2-aboundance on GBM cell lines and the MSCs capability to interact via GD2 tCAR surface expression. GD2 tCAR MSCs and bi-functional MSCs bound the highly GD2-positive T98G line at least four times more than EV MSCs and mTRAIL MSCs (*p* < .001; Fig. 4b). A lower but significant binding gain of GD2 tCAR MSCs and bi-functional MSCs was found for U87MG GD2-intermediate line (*p* < .01; Fig. 4c). As for the A172 GD2-negative line, no differences in aggregate formation were detected among MSC groups, as this tumor cell line does not express GD2 ligand (Fig. 4d). As a further confirmation, GD2 tCAR MSCs and bi-functional MSCs led to a greater binding to primary C3c GBM line in comparison to EV MSCs and mTRAIL MSCs (*p* < .05; Fig. 4e). Collectively, these findings indicate that MSCs functionalized by an anti-GD2 AR gain an increased and specific binding to GBM cells expressing different levels of GD2.

### Co-expression of mTRAIL and GD2 tCAR confers to bi-functional MSCs a rapid killing activity against GD2-positive GBM cells

**Fig. 5:**
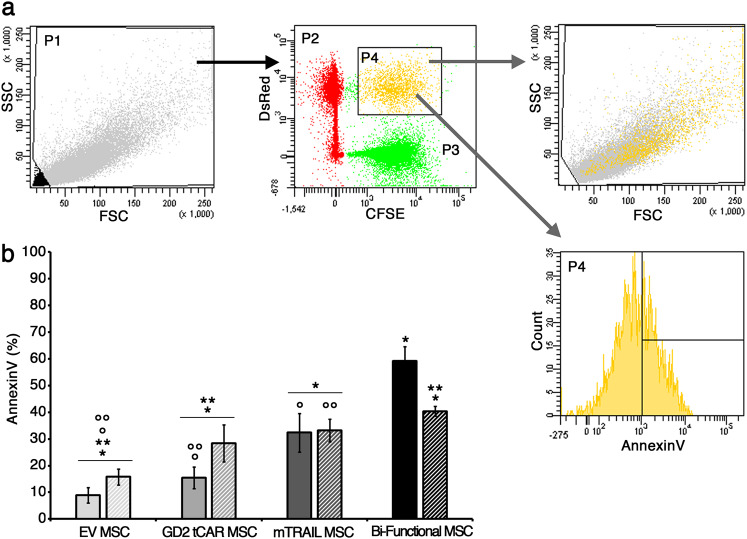
Bi-functional MSCs exert specific in vitro cytotoxicity at an early time point against T98G GBM cell line by a cell-to-cell interaction cytotoxicity assay. **a** Gating strategy applied to quantify the percentage (%) of Annexin V-positive MSC-GBM cell aggregates after 7 h incubation time. Gate 1: forward scatter (FSC) and side scatter (SSC) were used to enrich for intact cells (P1). Gate 2: CFSE staining (P3) and DsRed fluorescence (P2) recorded in the logarithmic scale were used to identify MSC-GBM cell aggregates visualized as CFSE/DsRed double positive (P4). Backgating in P1 population to identify MSC-GBM cell aggregates by morphological parameters. Early cell death in MSC-GBM cell aggregates was evaluated by Annexin V (APC) by FACS gating on CFSE/DsRed-double-positive conglomerates while recording a fixed number of events for all conditions. **b** Interaction between bi-functional MSCs and GBM cells (smooth columns) produced a significant apoptotic effect on cell aggregates (59 ± 5%), compared to both mTRAIL MSCs (32 ± 7%) and non-expressing TRAIL ones, EV MSCs (9 ± 3%) and GD2 tCAR MSCs (15 ± 4%) (*p < .001). Blocking GD2 antigen–GD2 tCAR binding by anti-idiotype sera incubation (striped columns), significantly reduced the percentage of Annexin V-positive aggregates (40 ± 2%), comparable to mTRAlL MSCs with (36 ± 7%) or without (32 ± 7%) sera incubation. *p < .001, **p < .001, °*p* < .001, and °°*p* < .001 by Student’s *t* test. Data are expressed as mean ± SD

A cell-to-cell interaction cytotoxicity assay was performed to address whether the enhanced binding ability of bi-functional MSCs to GD2-positive GBM cells would translate into a more effective killing compared to mTRAIL MSCs. Cell death was evaluated at early time point (7 h) by Annexin V on CFSE/DsRed double-positive aggregates. A gating strategy (Fig. 5a) was applied also to this experimental setting and the percentage of Annexin V-positive MSC-GBM cell aggregates (P4) was quantified. To confirm the GD2 tCAR role in leading a more rapid apoptosis mediated by bi-functional MSCs, anti-idiotype mice sera against the GD2 tCAR were used to block the binding between GD2 ligand and GD2 tCAR (Fig. 5b). Interaction between bi-functional MSCs and GBM cells produced a significant apoptotic effect on cell aggregates (59 ± 5%), compared to both mTRAIL MSCs (32 ± 7%; *p* < .001) and non-expressing TRAIL ones, EV MSCs and GD2 tCAR MSCs (9 ± 3% and 15 ± 4%, respectively, *p* < .001; Fig. 5b, smooth columns).

Blocking the GD2-mediated interaction by anti-idiotype sera incubation [18, 24], bi-functional MSCs significantly reduced the percentage of Annexin V-positive aggregates (40 ± 2%), comparable to mTRAIL MSCs with (36 ± 7%) or without sera incubation (32 ± 7%; *p* > .05; Fig. 5b, striped columns). These data suggest that GD2 tCAR expression on bi-functional MSCs allows a specific targeting to GD2-expressing GBM potentiating the cytotoxic effect.

## Discussion

We have here explored how engineered human MSCs by a truncated IgM-derived anti-GD2 CAR can be a powerful new tool to specifically redirect MSCs delivering TRAIL against a GD2-expressing tumor, such as GBM. The invasive nature of GBM is still the major reason for failure in surgical resection and after standard therapeutic approaches [5]. The tumor-tropic ability of MSCs offers an alternative where these cells can be used as vehicle of anti-tumor molecules delivery to modify tumor microenvironment and destroy cancer from inside, the so-called “Trojan horse effect” [1]. MSCs can home and persist into the tumor inflammatory stroma arranging themselves in close proximity with tumor cells and neovasculature [14]. Among anti-cancer drugs deliverable by MSCs there is the protein known as TRAIL. To date, multiple attempts to use MSCs as TRAIL-vehicle against GBM have been described [25, 26]. However, even though MSCs tropism for tumors has been confirmed in a variety of experimental models, there is no evidence of an efficient and long-term engraftment of MSCs, highlighting that inherent MSCs homing and retention capabilities into the tissue of interest might be insufficient for solid therapeutic benefit [27]. Efforts should be therefore directed towards optimization of both efficacy and specificity of MSC-based therapy targeting a specific cell population and prolonging MSCs retention. While these tasks are challenging, MSC cancer-targeting strategies may be employed to enhance the efficiency in reaching out tumors and consequently increase the bioavailability of the therapeutics. These may include physical cell routing, utilization of physiological forces for cell concentration, and strategies that involve engineering cell homing/targeting machinery [15].

By multiple in vitro assays, we explored whether the native tumor tropism of MSCs can be enhanced by engineered targeting via expression of a tumor-binding AR. First, we investigated the feasibility of expressing the GD2 tCAR on the cell surface of adipose MSCs by lentiviral transduction, in order to specifically target GD2-expressing tumor cells. The transduction of AD-MSCs with the vector encoding for GD2 tCAR was very efficient and did not interfere with the simultaneous expression of TRAIL.

Previous works gave evidences of MSCs localization into glioma after systemic delivery or local intracranial injection followed by migration towards GBM [28, 29]. Once there, MSCs can also effectively deliver therapeutic proteins with a pre-clinical efficacy [6, 25, 26]. As reported, GBM represents one of the most GD2-positive solid tumor [16, 17, 30], suggesting that an anti-GD2-immunotargeted MSC-based strategy may further enhance their therapeutic profile.

**Fig. 6:**
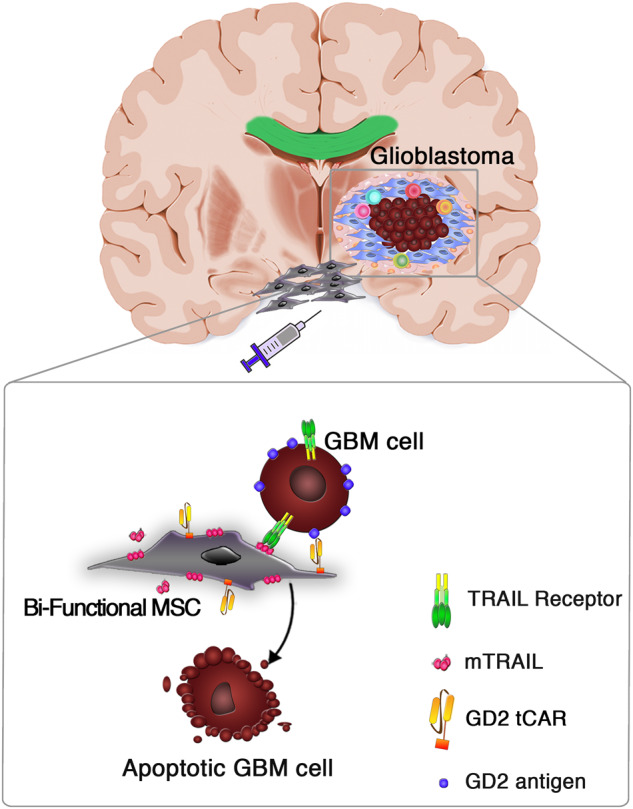
Bi-functional MSC-based targeting approach against GBM. Scheme representing the possible targeted killing mechanism by locally injected bi-functional MSCs co-expressing the truncated form of the anti-GD2 CAR (GD2 tCAR) and the membrane-bound TRAIL (mTRAIL). MSCs surface functionalization with GD2 tCAR could enforce the in vivo interaction with GD2-expressing GBM cells, allowing a selective target recognition in such a way to empower mTRAIL-mediated pro-apoptotic effect

Considering this MSCs tropism for brain tumors, we hypothesized that strengthening adherence to tumor cells by GD2 tCAR could enhance bi-functional MSCs retention within GD2-positive GBM tumor bed while potentially sparing the normal brain tissue. This in turn may affect the efficacy of our cell-based approach, as pre-requisite for a local delivery of bi-functional MSCs into GBM site (Fig. 6). Three different GBM cell lines were considered. GBM lines were first characterized in terms of GD2 antigen expression, as predictive factor for affinity-based targeting. Based on GD2 antigen abundance on the surface, we distinguished a GD2 highly-positive (T98G), a GD2-middle positive (U87MG), and a GD2-negative cell line (A172). We also considered a primary GBM sample providing evidence of a relevant GD2 expression, as previously reported [17].

All tested GBM cell lines were highly positive for the agonistic receptor DR5, implying a possible responsiveness to TRAIL apoptotic effect and did not express DcR1 and DcR2, exception made for U87MG where intermediate levels of DcR2 were detected, as previously described by Röhn et al. [31]. For instance, the primary C3c GBM line expressed high levels of DR5, whereas DR4 and both decoy receptors were undetectable. A dose-dependent effect of rhTRAIL protein was tested to confirm their sensitivity. As expected, rhTRAIL induced apoptosis in both T98G and A172, in a dose-dependent manner, whereas U87MG cells resulted less sensitive to apoptotic effect performed by rhTRAIL.

Looking at first to cytotoxicity, bi-functional MSCs effectively killed all GBM lines in coculture assay; however, the effect was stronger against T98G and A172 than U87MG line. We assume that the lower sensitivity displayed by U87MG could be due to different molecular mechanisms impacting on the TRAIL pathway which need to be better investigated. For instance, a study by Qiu et al. [32] indicates that paclitaxel, a member of a class of anti-cancer drugs known as taxanes, can sensitize U87MG cells to TRAIL by upregulating DR4, caspase-8 and caspase-3. Our group, in the attempt to overcome the U87MG partial resistance towards TRAIL-induced cell death, recently sensitized U87MG line by paclitaxel treatment, reaching successful levels of apoptosis by mTRAIL MSCs (not shown). In addition, bi-functional MSCs proved to be effective also against the primary C3c line, further confirming that the simultaneous expression of pro-apoptotic TRAIL ligand and GD2 tCAR protein does not affect the killing by bi-functional MSCs.

In parallel to the assessment of cytotoxicity, we investigated whether the GD2 tCAR was capable to improve MSCs affinity for GBM, hence extending TRAIL-mediated therapeutic effect and limiting off-target toxicities. Thus, we have challenged bi-functional MSCs ability to bind GD2-expressing GBM cells in a cell-to-cell interaction assay demonstrating that the number of MSC-GBM cell aggregates, collected after interaction, strictly correlated with both GD2-aboundance on GBM cell lines and the MSCs capability to interact via GD2 tCAR surface expression. The bi-functional MSCs selectively bound the highly T98G and the middle U87MG GD2-expressing lines at a higher extent than mTRAIL MSCs. Similarly, by challenging bi-functional MSCs interaction with primary C3c cells, we found a significantly higher number of aggregates compared to mTRAIL MSCs. For the A172 GD2-negative line, no differences in aggregates formation were detected, suggesting that MSCs functionalization by the anti-GD2 AR is associated with increased and specific binding restricted to GD2-expressing GBM cells.

The cell-to-cell interaction cytotoxicity assay further demonstrated that the enhanced binding by bi-functional MSCs led to a more effective killing activity against GD2-positive T98G cells, compared to that mediated by mTRAIL MSCs. As a further confirmation, blocking the GD2-mediated interaction by bi-functional MSCs incubation with mice sera against GD2 tCAR restored a killing potential comparable to that observed with mTRAlL MSCs. These data demonstrated that GD2 tCAR is able to force the interaction between bi-functional MSCs and GBM-expressing GD2 antigen, potentiating the mTRAIL-mediated cytotoxic effect owing to a selective binding to GD2-positive GBM cells. Nevertheless, an optimization within a relevant animal model shall be necessary in order to better take advantage of the specific GD2 tCAR-binding capability to the GD2 antigen and to evaluate the therapeutic benefits of bi-functional MSCs carrying both GD2 tCAR and TRAIL.

Targeted delivery of therapeutics has been so far restricted to lymphocytes based on CAR or bispecific adaptors [15]. Recently, affinity-based cell targeting has been also applied to MSCs. Tumor-specific targeting and retention were achieved by genetic modification of MSCs with AR against EGFRvIII in GBM [14] and erbB2 in ovarian cancer [19]. Balyasnikova et al. [14] investigated the feasibility of expressing a scFv against EGFRvIII on the surface of MSCs using nucleofection, in order to specifically target GBM cells expressing EGFRvIII. Similarly, Komarova et al. [19] created MSCs that express an AR that binds to erbB2, to explore the possibility of increasing the number of MSCs in ovarian tumors. Regarding CAR technology applied to target TRAIL receptors, Kobayashi et al. [33] generated a CAR with a TRAIL-R1-specific scFv antibody (TR1-scFv-CAR) transferring it on several cell types, including human peripheral blood lymphocytes (PBLs). They report that the TR1-scFv-CAR-expressing PBLs in vitro killed target cells not only via TR1-mediated apoptosis but also by CAR signal-induced cytolysis. However, in these findings there is no evidence of an anti-cancer molecule delivery by cells simultaneously expressing an AR. Taking inspiration from CAR T cells, for the first time to our knowledge, we coupled the affinity and the cytotoxicity by anti-GD2 and TRAIL dual-targeting delivered using human mesenchymal progenitors. This CAR-based therapeutic approach holds the promise to achieve site-specific and prolonged retention of MSCs to the tumor site, thereby providing an effective delivery of TRAIL pro-apoptotic molecule in the context of GD2-expressing cancer.
